# Empirical study on the factors influencing the successful aging of the middle-aged and older adult community volunteers

**DOI:** 10.3389/fpubh.2023.1140965

**Published:** 2023-10-04

**Authors:** Chien-Chih Chen, Yu-Li Lan, Yu-Hua Yan

**Affiliations:** ^1^Department of Future Studies and LOHAS Industry, Fo Guang University, Yilan, Taiwan; ^2^Department of Health Administration, Tzu Chi University of Science and Technology, Hualien, Taiwan; ^3^Superintendent Office, Tainan Municipal Hospital (Managed by Show Chwan Medical Care Corporation), Tainan, Taiwan

**Keywords:** middle-aged and older adult, volunteering, participation motivation, expectation confirmation, successful aging

## Abstract

**Background:**

The pursuit of successful aging is currently the most important research and policy issue in an aging society. Participating in voluntary services can help middle-aged and older adults recognize the positive value and benefits of social participation, feel a sense of happiness and accomplishment, and improve their overall life satisfaction, which can also contribute to successful aging. This study wants to understand whether the participation motivation and expectation confirmation of middle-aged and older adult volunteering will affect their continuous participation behavior and successful aging because of the satisfaction of actual participation?

**Objective:**

This study explores the factors related to middle-aged and older adult volunteering participation and their impact on successful aging.

**Methods:**

Middle-aged and older adult volunteering from the East Taiwan Community Development Association and community care centers were taken as the research objects. Convenience sampling was used to select volunteers who were over 45 years old (inclusive) and have participated in voluntary services over five (inclusive) times in the last 6 months. Respondents completed the questionnaire through self-completion or face-to-face interviews with the interviewer. The measurement tools include engagement motivation, expectation validation, satisfaction, ongoing engagement, and successful aging.

**Results:**

A total of 536 questionnaires were distributed of which 498 were valid and 38 invalid. The questionnaire recovery rate was 92.91%. Statistical findings include: (1) Those who perceived that their health was good had a better successful aging status than those who perceived that their health was normal. (2) The volunteering participation motivation and expectation confirmation of middle-aged and older adults significantly affected their volunteer participation satisfaction. (3) Participation motivation and expectation confirmation predicted 50.8% of satisfaction. (4) Satisfaction predicted 47.1% of continuous participation. (5) Continuous participation and satisfaction had a predictive power of 65.1% for successful aging.

**Conclusion:**

This study confirms that the motivation and expectation of middle-aged and older adult to participate in volunteering will affect their continuous participation behavior and successful aging status through satisfaction. The research results can be used as a reference for the practical work plan of volunteering.

## 1. Introduction

Population aging is a global trend. The United Nations predicts that the rate of population aging in the 21st century will surpass that of the 20th century (1). In 2018, Taiwan officially entered an aging society (the older adult population accounted for over 14.6% of the total population), rising to 15.2% by November 2019, and is expected to enter a super-aging society (over 20.1%) by 2025 (2).

In an aging society, promoting successful aging can protect older adults from illness and disability, reduce the social governance burden and welfare delivery cost, and enable them to enjoy their old age (3). Successful aging refers to realizing one’s potential and achieving a level of physical, social, and mental health in later life that is beneficial to oneself and others (4). Rowe and Kahn (5) defined three elements of successful aging: (1) reducing the incidence of disease and disability, (2) maintaining a high degree of cognitive and physical function, and (3) actively participating in daily activities. Successful aging involves maintaining a state of vigor and health in later years of life. For discussions on successful aging, relevant research includes: Von Faber et al. (6) uncovered that successful aging has multiple dimensions, including physical functioning, social functioning, psycho-cognitive functioning, and well-being. Phelan and Larson (7) summarized several studies on successful aging, including life satisfaction, longevity, freedom from disability, mastery/growth, active engagement with life, high/independent functioning, and positive adaptation. Chou and Chi (8) divided successful aging into four dimensions: physical function, emotional state, cognitive function, and productivity. Therefore, the pursuit of successful aging is an unavoidable and urgent task in an aging society, which is also currently the most important research and policy issue (9, 10).

The health concerns of older adults are no longer limited to chronic diseases or long-term disabilities but also include how to promote the health of older adults while prolonging life and minimizing mortality risk. This not only includes reducing diseases and disabilities but also maintaining good physical and mental functions, further promoting social and psychological health, and enabling older adults to actively enjoy their old age (11). Older adults can benefit from volunteering (12–16). Participants who volunteer can stay connected with others, be productive in their activities, and avoid illness and disability (17). Volunteering can help meet these needs and allow older adults to better adapt to the aging process (16). Volunteering can help older adults maintain their social skills, participate in society, and contribute to their physical and psychological health to achieve successful aging (5). A systematic review of the current literature found that volunteering among international older adults was associated with reduced depressive symptoms, improved health, fewer functional limitations, and lower mortality (18). Volunteering can help older adults recognize the positive value and feedback from social participation, feel a sense of happiness and accomplishment, and thus improve overall life satisfaction (19). Therefore, volunteering is considered an effective health promotion program for older adults as it can substantially improve their health status (20).

Older adults must be motivated to participate in volunteering. Liu Yiling (21) summarized the literature on self-interested motivation, altruistic motivation, social motivation, and situational factor motivation. Expectation Confirmation Theory (ECT) (22) is an extended application research on the theoretical model of satisfaction cognition. It can be used to measure the gap between the user’s or provider’s expected cognition and actual cognition. Satisfaction with volunteering can be measured by identifying expected cognitions before volunteering and cognitions after actual service experience. The level of satisfaction will affect the willingness to participate in the service in the future.

This study is based on the premise that middle-aged and older adults participation in volunteering positively impacts their successful aging, and middle-aged and older adult’s volunteer participation must be motivated to encourage participation, action, commitment, and contribution. Therefore, this study aimed to understand the motivation factors for middle-aged and older adults to participate in volunteering. Middle-aged and older adults have expectations about the content of the service before volunteering. However, expectations before the service may differ from actual emotions after the service. Therefore, this study also aimed to determine the expectations and affirmations of middle-aged and older adults who participate in volunteering. In addition, the participation motivation and expectation confirmation of middle-aged and older adults participating in volunteering may affect their continuous participation behavior and successful aging through the satisfaction they perceive after the actual service. Thus, this study explores whether the two factors above will affect their continuous participation behavior and successful aging owing to their satisfaction with the actual participation.

## 2. Materials and methods

### 2.1. Study design and participants

In this study, a cross-sectional survey method was used to understand the factors related to middle-aged and older adults participation in community volunteering and their impact on successful aging. Middle-aged and older adult volunteers of the Eastern Taiwan Community Development Association and community care bases were included as research participants. A convenience sampling method was adopted with the following sampling conditions: those who were over 45 years old (inclusive) and had participated in voluntary services more than five times (inclusive) in the last 6 months; those who can communicate in Chinese, Taiwanese, and Hakka; and those who agreed to participate in this research. Approval was obtained from the Research Ethics Committee of Hualien Tzu Chi Hospital, Buddhist Tzu Chi Medical Foundation (IRB111-054-B).

### 2.2. Research framework and hypotheses

**Figure 1:**
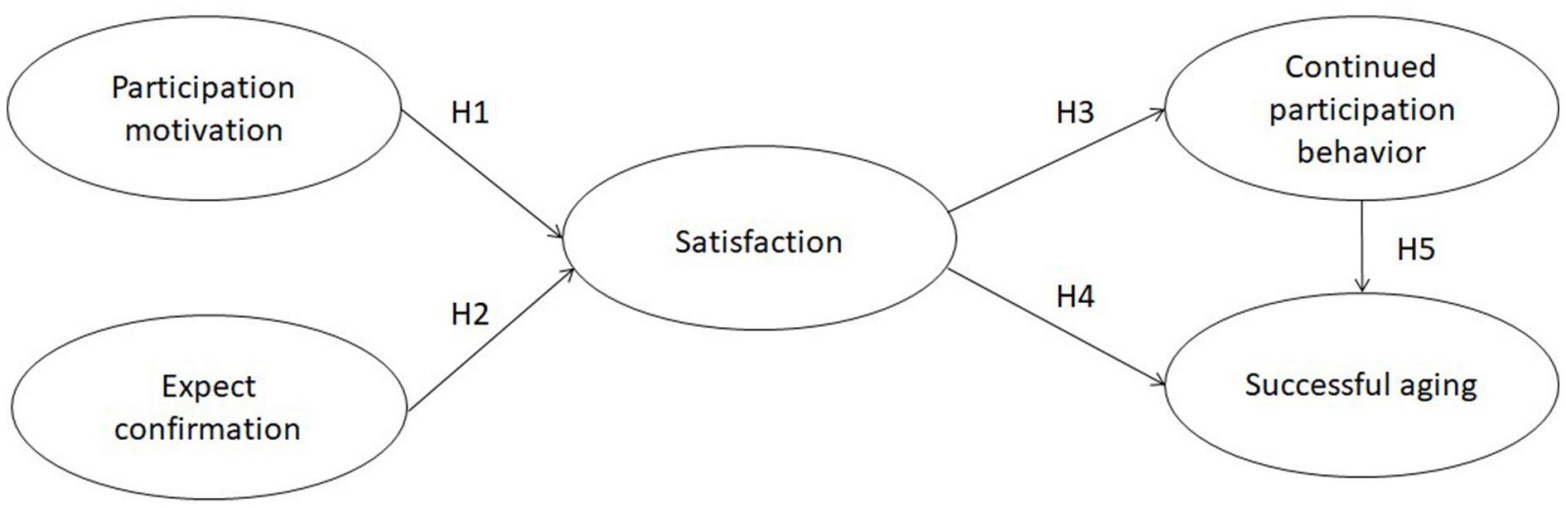
Research framework.

The following hypotheses are proposed on the basis of the research framework (see Figure 1):

*H1*: Participation motivation is positively related to satisfaction. The higher the motivation of middle-aged and older adults to participate in voluntary service, the greater their satisfaction.

*H2*: Expectation confirmation is positively related to satisfaction. The higher the expectation and affirmation of middle-aged and older adults in volunteer participation, the greater their satisfaction.

*H3*: Satisfaction is positively related to continued participation behavior. The higher the satisfaction of middle-aged and older adults in volunteer participation, the higher their continued participation behavior.

*H4*: Satisfaction is positively related to successful aging. The higher the satisfaction of middle-aged and older adults in volunteer participation, the better their successful aging.

*H5*: Continue to participate behavior is positively related to successful aging. The more middle-aged and older adults continue to participate in volunteering, the better their successful aging will be.

### 2.3. Research tools

A structured questionnaire was adopted in this study. Five experts and scholars from related fields were invited to conduct expert validity analysis through a pre-test of 30 questionnaires. The questionnaires were scored according to importance, clarity and appropriateness. The Content validity index (CVI) of expert validity was above 0.85.

Measurement tools included the following: (1) participant demographics; (2) participation motivation from a total of 25 items based on relevant literature (23–25) including: influencing others (6 items, *Cronbach’s α* = 0.880), social responsibility (6 items, *Cronbach’s α* = 0.942), self-growth (8 items, *Cronbach’s α* = 0.957), and interpersonal relationship (5 items, *Cronbach’s α* = 0.881); (3) expectation confirmation (5 items, *Cronbach’s α* = 0.945) (26); (4) satisfaction (4 items, *Cronbach’s α* = 0.933) (26); (5) continuous participation behavior (4 items, *Cronbach’s α* = 0.923) in volunteering (26); (6) successful aging from a total of 25 items (5, 7) was divided into physiology (5 items, *Cronbach’s α* = 0.929), psychology (10 items, *Cronbach’s α* = 0.948), and society (10 items, *Cronbach’s α* = 0.942). The answers were scored on a five-point Likert scale (1 being strongly disagree and 5 being strongly agree), with higher scores representing higher agreement.

### 2.4. Data collection

Ten community development associations and community care bases were preset by the researchers, and consent and approval of the chairman and relevant cadres were obtained through a phone call. The participants were instructed to come to the institution upon the agreed date and time. Thereafter, the research purpose and related information were also provided. Consent was obtained from the participants prior to their survey participation. Respondents completed the questionnaire through self-completion or face-to-face interviews with the interviewer (the interview duration was 15–20 min). Participants were compensated 100 yuan for their survey participation. They were also informed that the results of the data collection would remain confidential.

### 2.5. Data analysis

Statistical analysis was performed using SPSS 22.0. The distribution of the background data of the research object was represented using percentages, average values, and standard deviations. The difference in successful aging in different demographic background variables was analyzed using *t*-test and one-way variance analysis. If the variance analysis found a significant difference, Scheffe’s post-hoc test was performed. Structural equation modeling (SEM) path analysis was conducted using AMOS 5.0 statistical software.

## 3. Results

### 3.1. Demographic characteristics

A total of 536 questionnaires were distributed in this study, of which 498 were valid and 38 invalid, this included those who did not complete the questionnaire or questionnaire interview. The return rate for the questionnaire was 92.91%. Approximately 362 (72.7%) were women. Regarding education level, 203 (40.8%) had high school level education, followed by 106 (21.3%) with university-level education. Most were married 385 (77.3%), and 174 (34.9%) lived with their spouses. For most, economic status was balanced, accounting for 365 (73.3%); 269 (54.0%) perceived that they were in good health. Most had volunteered for over 6 months to 5 years, followed by 102 (20.6%) who had volunteered for 6 to 10 years (Table 1).

**Table 1:** Frequency distribution (*N* = 498).

Variable	Variable label	Frequency	Percentage	Cumulative percentage
Sex	Male	136	27.3	27.3
	Female	362	72.7	100.0
Education level	Elementary school	95	19.1	19.1
	Secondary school	81	16.3	35.3
	High school/vocational training	203	40.8	76.1
	University/College	106	21.3	97.4
	Institute (including) and above	13	2.6	100.0
Marital status	Single	40	8.0	8.0
	Married	385	77.3	85.3
	Divorced	65	13.1	98.4
	Widowed	8	1.6	100.0
Residence type	Live alone	95	19.1	19.1
	Live with spouse	174	34.9	54.0
	Live with children	83	16.7	70.7
	Live with children and spouse	146	29.3	100.0
Economic status	Not enough	22	4.4	4.4
	Balance of payments	365	73.3	77.7
	Have surplus	111	22.3	100.0
Perceived health	Not good	20	4.0	4.0
	Ordinary	209	42.0	46.0
	Good	269	54.0	100.0
Volunteer years	0.5–5 years	168	34.0	34.0
	6–10 years	102	20.6	54.7
	11–15 years	66	13.4	68.0
	15–20 years	76	15.4	83.4
	Over 20 years	82	16.6	100.0

### 3.2. Descriptive analysis of each scale (1–5 scoring)

Participation motivation was dealt with within 25 questions and had an average score of 4.36. The highest score was “social responsibility,” which included six questions with an average score of 4.41 points; “interpersonal relationship” included five questions with average score of 4.40 points; “self-growth” had eight questions with an average score of 4.37 points; the influence of others had six questions with an average score of 4.28 points. Expectation confirmation comprised five questions with an average score of 4.37. Satisfaction included four questions with an average of 4.46 points. Continuous participation had four questions with an average score of 4.32. Successful aging included 25 questions with an average score of 4.37 points. Societal included ten questions and was highest with an average of 4.42 points; it was followed by ten psychological questions, with an average score of 4.37 points; and five psychological questions, with an average score of 4.28 points (Table 2).

**Table 2:** Mean and standard deviation of construct.

Construct	Item	Number of questions	Means	Std. Deviation
Participation motivation	Influenced by others	6	4.28	0.54
	Social responsibility	6	4.41	0.52
	Self-growth	8	4.37	0.53
	Interpersonal relationship	5	4.40	0.48
	Total	25	4.36	0.47
Expect confirmation		5	4.37	0.52
Satisfaction		4	4.46	0.52
Continued participation behavior		4	4.32	0.54
Successful aging	Physiological	5	4.28	0.55
	Psychological	10	4.37	0.48
	Societal	10	4.42	0.48
	Total	25	4.37	0.46

### 3.3. Independent samples’ *t*-test and ANOVA test

The difference in successful aging in different demographic backgrounds was tested using the *t*-test, and no statistically significant difference was found. The difference between successful aging in different demographic background variables was analyzed through one-way variance analysis. If the variance analysis found a significant difference, Scheffe’s post-hoc test was performed. The results of the study demonstrated a statistically significant difference in the “perceived health” of successful aging (*p* < 0.05). Further, Scheffe’s post-hoc test unveiled that the successful aging status of those who perceived that their health was good was higher than that of those who perceived that health was normal (*p* < 0.05) (Table 3).

**Table 3:** Differences in demographic variables in each ANOVA construct.

Construct	Perceived health	*N*	Mean	Std. Deviation	F-test	Value of *p*	Scheffe
Successful aging	Not good	20	4.67	0.579	3.590	0.028*	Good > Ordinary
	Ordinary	209	4.71	0.538			
	Good	269	4.83	0.458			

### 3.4. Path coefficient analysis variables for SEM verification of causality

**Figure 2:**
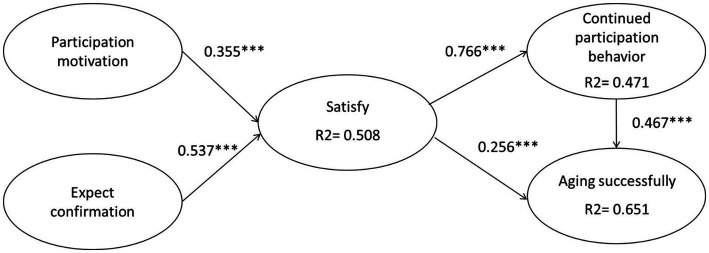
SEM statistic model.

Participation motivation and expectation confirmation significantly affected satisfaction (*β* = 0.355***; *β* = 0.537***); thus, H1 and H2 were supported (Figure 2). Satisfaction significantly affected sustained engagement and successful aging (*β* = 0.766***; *β* = 0.256***); thus, H3 and H4 were supported. Sustained engagement significantly affected successful aging (*β* = 0.467***); thus, H5 was supported. The *R*^2^ test was also carried out in this study, and the results demonstrated that the predictive power of participation motivation and expectation confirmation for satisfaction reached 50.8%, the predictive power of satisfaction for continuous participation reached 47.1%, and the predictive power of continuous participation and satisfaction on successful aging reached 65.1% (Table 4).

**Table 4:** Direct effect analysis results.

Mode	Mode (Direct mode)				*R* ^2^
Hypothesis	Standard path coefficient		Significance (value of *p*)	Hypothesis verification	
H1	Participation motivation → Satisfaction	0.355	*p* < 0.000	Accept	0.508
H2	Expect confirmation → Satisfaction	0.537	*p* < 0.000	Accept	
H3	Satisfaction → Continued participation behavior	0.766	*p* < 0.000	Accept	0.471
H4	Satisfaction → Successful aging	0.256	*p* < 0.000	Accept	0.651
H5	Continued participation behavior → Successful aging	0.467	*p* < 0.000	Accept	

## 4. Discussion

This study is based on the premise of promoting healthy and successful aging of middle-aged and older adults in three aspects: psychology, physiology, and society. By exploring the participation motivations and expectations of middle-aged and older adult volunteers, we can determine whether the satisfaction entailed by volunteering can improve their continued participation behavior and promote successful aging. The results of this study found that the stronger the motivation and the higher the confirmation of expectations for middle-aged and older adults to participate in volunteering, the better their satisfaction with volunteering. The higher the satisfaction of middle-aged and older adults participating in volunteering, the higher their willingness to continue volunteering, and the greater the impact on their successful aging.

In this study, middle-aged and older adult volunteers have high scores in physical, psychological and social aspects of successful aging, and the motivation of middle-aged and older adult volunteers to participate in volunteering affects their successful aging through satisfaction. Volunteering is the most direct way and the main channel of social participation. By participating in voluntary services, middle-aged and older adults can play meaningful social roles, maintain social participation when providing services, establish new interpersonal relationships, and reduce feelings of isolation. Social participation is an indicator of successful aging, and continuous social participation significantly and positively impacts life, psychology, and health (27, 28). Some studies indicated that people increasingly recognize that social participation is related to successful aging (29) given that participating in social activities can promote physical health (30), mental health (31), intellectual function (32), and longevity (33). In addition, social activities improve physical function, are linked to lower risk of future dependence, and are associated with functional recovery and enhanced ability to perform daily activities (34). Although volunteering can benefit the health and well-being of older adults, we often overlook that it can allow older adults to better adapt to the aging process (16, 23). Related studies have also revealed that older adult volunteers have a higher sense of well-being than older adults who are non-volunteers (35). Volunteers experience fewer depressive symptoms and lower healthcare utilization than non-volunteers (36). Volunteering among older adults predicts improved self-rated health, physical function, and physical activity (37, 38).

In this study, the higher the confirmation of the expectations of the middle-aged and older adult volunteers, the higher the satisfaction. Higher satisfaction with volunteering is associated with greater willingness to continue volunteering and better aging success. Engaging older adult volunteers in social activities can enhance their social engagement and interaction, which can improve their overall well-being (39). Particularly, voluntary service has a positive meaning for middle-aged and older adults. Participating in volunteer work can help older adults enhance their self-worth and maintain a sense of connection with society. Participating in volunteer work can improve the mental health of older adults (13). Participants can stay connected to others and be productive during these activities; this helps them avoid illness and disability (5, 17) and obtain psychological and social benefits (40). This study demonstrates that the successful aging rate of those who perceive good health is higher than that of those who perceive normal health. Similar to the findings of previous studies, volunteering is associated with perceived health (41). In addition, results from previous longitudinal studies and randomized controlled trials with volunteers predict enhanced self-esteem and assessment of health, physical function, and physical activity (6, 38).

In this study, volunteer participation motivation and expectation confirmation of older adults significantly impacted their volunteer satisfaction, and the predictive power of participation motivation and expectation confirmation on satisfaction reached 50.8%. Satisfaction had an explanatory power of 47.1% for continuous participation. Continuous participation and satisfaction can explain 65.1% of successful aging. This indicates that the higher the participation motivation and expectation confirmation of middle-aged and older adult volunteers, the greater their satisfaction, and the better their continuous participation and successful aging. Sustained volunteerism also affects their successful aging status. Related studies have also uncovered that volunteer satisfaction is positively correlated with willingness to stay. Volunteering satisfaction significantly impacts behavioral intention. If an organization can consider the personalities and preferences of volunteers to arrange work content, it will reduce the employment rate and improve satisfaction (42, 43).

This study found that “social responsibility” is the highest motivation for middle-aged and older adult volunteers, followed by “interpersonal relationship” and “self-growth.” This reveals that middle-aged and older adults value the professional knowledge or skills they have learned to help others by participating in voluntary services. Middle-aged and older adults are willing to give back to social needs, establish and develop interpersonal relationships, and learn from them by participating in voluntary work. Similar to previous studies, the main factors motivating older adults to participate in volunteering are altruism (concern for others) and self-interested ideas (hoping to gain some benefits from this participation, including filling time, feeling useful and needed, increasing social identity, and improving self-esteem) (44). Related research also has a similar view; although altruism only emphasizes the needs of others, older adult volunteers may also benefit from this kind of activity (13). However, the results of this study also found that the participation motivation of middle-aged and older adult volunteers is not a single reason but comprises multiple reasons. Even if they participate in voluntary services for self-interest, they regard helping others as an obligation with social value. Therefore, the promotion and supervision organizations of volunteer groups can actively provide incentives and support measures and improve people’s sense of accomplishment in participating in voluntary services to stimulate participation motivation and meet their expectations. Simultaneously, establishing a perfect system and shaping a good organizational culture will effectively recruit new volunteers and maintain older adult volunteers.

## 5. Research limitations

Due to funding and time constraints, this study employed a cross-sectional research. Thus, more empirical research on the influence of middle-aged and older adults’ motivation and expectations for volunteering on successful aging is warranted. In addition, this study adopted convenience sampling for data collection; thus, conducting a survey on all community older adults was not possible, which may have limited the generalizability of the findings.

## 6. Conclusion

This study confirms that the motivation and expectation of middle-aged and older adults to participate in volunteering will affect their continuous participation behavior and successful aging status through satisfaction. With the aging of the world’s population, strengthening the social participation of older adults has become a key factor for successful aging that the government must prioritize. If the older adults can self-recognize the positive value and rewards of participating in volunteering, they will feel a sense of happiness and accomplishment, which can improve their overall life satisfaction and become productive seniors.

While strengthening older adults’ volunteering motivation, governments and NGOs should consider their health status, interests, and preferences for volunteering. Connecting volunteers to the activities they find meaningful and appropriate plays an important role. This study can serve as a reference for the practical work plan of volunteering and help the government and related organizations in gaining a thorough understanding of the motivation, expectation confirmation, and successful aging of middle-aged and older adult volunteers.

### 6.1. Recommendations for future research

It is recommended that follow-up research is conducted, including long-term observational studies. This would provide insight into the impact on the continuum of middle-aged and older volunteers and possible issue. For example: explore the preferences of middle-aged and older adults to participate in volunteering? How to improve the satisfaction and multiple influencing factors of middle-aged and older adults? How to improve the satisfaction of middle-aged and older adults and its multiple influencing factors? It can also explore the content or mode of operation suitable for middle-aged and older adults to participate in volunteering, or the impact of different attributes of volunteer work on their successful aging. It will help improve the self-esteem, sense of accomplishment and life satisfaction of middle-aged and older adults, and enable them to play their unique advantages in community organization animation.

## Data availability statement

The original contributions presented in the study are included in the article/supplementary material, further inquiries can be directed to the corresponding authors.

## Ethics statement

The studies involving human participants were reviewed and approved by Ethical Review Committee of the Hualien Tzu Chi Hospital, Buddhist Tzu Chi Medical Foundation (IRB111-054-B). The patients/participants provided their written informed consent to participate in this study.

## Author contributions

C-CC and Y-LL conceived and designed the study and drafted the manuscript. C-CC, Y-LL, and Y-HY participated in the acquisition of data and revised the manuscript.Y-LL and Y-HY analyzed the data. All authors contributed to the article and approved the submitted version.
